# European Union Cohesion Policy: Socio-Economic Vulnerability of the Regions and the COVID-19 Shock

**DOI:** 10.1007/s11482-022-10116-1

**Published:** 2022-11-18

**Authors:** Angeles Sánchez, Eduardo Jiménez-Fernández

**Affiliations:** 1 Department of Applied Economics, Faculty of Economics, Campus Cartuja, S/N, Universidad de Granada, 18071 Granada, Spain; 2 Department of Economics Theory and History, Faculty of Economics, Campus Cartuja, S/N, Universidad de Granada, 18071 Granada, Spain

**Keywords:** Cohesion policy, composite indicator, government expenditure, multilevel modeling, socio-economic vulnerability, sustainable development

## Abstract

The European Union Cohesion Policy for the period 2021–2027 focuses on five goals to make the European Union smarter, greener, more connected, more social and closer to citizens. However, a macroeconomic index is proposed as the predominant criterion for allocating the Structural Funds among regions. In this paper, we hypothesise that it is possible to take into account new, complementary criteria that better reflect citizens’ quality of life. To that end, we build a composite index of socio-economic vulnerability for the 233 regions. The results show that following our multidimensional approach for allocating the Structural Funds, there are remarkable differences in the maps of priority regions. In addition, the COVID-19 pandemic represents a threat to well-being. Are all regions equally exposed to COVID-19 in terms of their socio-economic vulnerability? To address this issue, we estimate multilevel models which indicate that country characteristics interact with regions’ characteristics to alter patterns of vulnerability. More specifically, increases in government expenditures in education and an improvement in political stability would reduce the regional vulnerability or foster the capacity for resilience, whereas increases in poverty would be associated with greater vulnerability. Likewise, more vulnerable regions would be the most exposed to the negative socio-economic effects of COVID-19. However, it is remarkable that several regions of Sweden and Finland would be among the group of regions whose socio-economic vulnerability would be the most negatively affected.

## Introduction

The main objective of the European Union (EU) Regional Policy, or Cohesion Policy, is to reduce the disparities between the levels of development of the regions and the backwardness of lagging regions. The EU Cohesion Policy for the 2021–2027 Multiannual Financial Framework aims at fostering a modernised regional development and cohesion policy focusing on five political goals so that the EU becomes: (1) smarter, through innovation and digitisation, (2) greener, (3) more connected, (4) more social and (5) closer to citizens (European Commission, 2018). The EU will dedicate 34% of its budget over 2021–2027 to cohesion and values, that is, economic, social and territorial cohesion and investment in competitiveness, people and values (European Commission, 2020a). This is the item that will receive the highest amount of commitment appropriations. Structural Funds are the main source of funding to implement the EU Cohesion Policy.

These guidelines represent significant challenges for the design of the regional development policies within the scope of ‘beyond GDP’, according to which the European Commission should develop several indicators that complement the gross domestic product (GDP) to support policy decisions through more comprehensive information (Commission of the European Communities, 2009). The EU opts for the increasingly accepted train of thought, stressing that GDP is insufficient to analyse the overall development and progress of society, and the measurement of regional development has to struggle with the multidimensional nature of well-being (O’Donnell et al., 2014; Stiglitz et al., 2018; Van den Bergh, 2009). However, a single macroeconomic index is again proposed as the predominant criterion for allocating the Structural Funds among the regions in 2021–2027.

In this paper, we hypothesise that new complementary criteria could be taken into account in line with the five goals of EU Cohesion Policy outlined above in order to better reflect the reality on the ground of the regions. With this in mind, the first aim of this paper is to construct a composite indicator of socio-economic vulnerability (SEVI) that synthesises the position of each EU region (NUTS-2 of the 27 Member States) in 2017 with respect to the five goals of the EU Cohesion Policy for 2021–2027.^1^

In addition, the COVID-19 pandemic caused by the SARS-CoV-2 coronavirus represents a threat to people’s well-being and new public policy challenges. Worldwide, the COVID-19 pandemic is a serious threat to the achievement of the Sustainable Development Goals since it is pushing tens of millions of people back into extreme poverty, putting years of progress at risk (United Nations, 2020a). In the context of the EU, it is foreseeable that COVID-19 will negatively affect the socio-economic development of the regions, as well as the quality of life of people since COVID-19 is impacting on a wide range of aspects: health and subjective well-being, social capital, human capital, product markets, financial markets and public finance (Bittmann, 2021; Bonaccorsia et al., 2020; Fasani & Mazza, 2020; Fetting, 2020; Giovanis & Ozdamar, 2022; Giovannini et al., 2020; Shek, 2021; United Nations, 2020b).

Faced with this situation, in its first annual strategic foresight report, the European Commission describes the first lessons of the COVID-19 crisis and introduces resilience as a new compass for the development of EU policies (European Commission, 2020b). In the report, the European Commission presents resilience dashboards in the socio-economic, green and digital dimensions and proposes further discussion to explore the feasibility of developing a synthetic resilience index. At the financial level, the EU has approved the Next Generation EU (Euro 750 billion) to build a more resilient, sustainable and fair Europe through large-scale financial support for investment and reforms. The majority of funds (Euro 672.5 billion) will be allocated to the Recovery and Resilience Facility programme to support public investments and green and digital projects in the crucial first years of the recovery after the pandemic.

In this scenario, assessing how changes in the environment or covariates of the regions could affect their socio-economic vulnerability is key for the planning of Cohesion Policy in order to determine actions that can increase the resilience of different territories. Accordingly, the second aim of this paper is to check whether country characteristics interact with regions’ characteristics to alter patterns of vulnerability. That is, we check if the structure of regions’ socio-economic vulnerability is hierarchical and causes a ‘country effect’ or if the socio-economic vulnerability of the regions differs across countries. If this interaction or country effect were confirmed, the third aim of this paper would be to analyse both the idiosyncratic and covariate shocks that COVID-19 might represent for the vulnerability of EU regions. In the context of the EU, formulating objectives 2 and 3 is highly significant due to the existence of a multilevel governance system with central, state and local governments in most of the Member States that assume different competences in matters of public policy affecting citizens’ quality of life. If the hierarchical structure were confirmed, multilevel modelling would be a suitable approach to address these two aims since standard estimation techniques could lead to incorrect conclusions (see Goldstein, 2011; Snijders & Bosker, 2012).

**Fig. 1:**
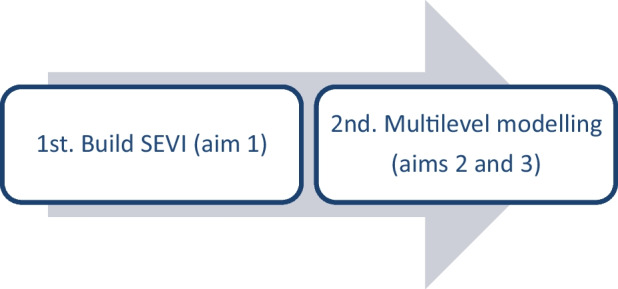
Two stages in the study of socio-economic vulnerability of the EU regions

To sum up, this paper aims to achieve three objectives. Firstly, in a first stage, we build a composite indicator to study the socio-economic vulnerability of the EU regions in terms of the 2021–2027 Cohesion Policy. Once we have an instrument (SEVI) to analyse the socio-economic vulnerability of EU regions, in a second stage we estimate mixed effects models or multilevel models with SEVI as the dependent variable, which allows us to achieve aims 2 and 3. Figure 1 indicates the two stages of our work.

The main contributions of our paper are twofold. First, we take the concept of vulnerability from other fields, such as poverty and economics, where it is studied at the individual level, and apply it to the level of regions. To this end, we follow a multidimensional approach to identify the factors driving socio-economic fragility and resilience in terms of the 2021–2027 EU Cohesion Policy goals and take into account findings from the first studies on the social and economic effects of COVID-19. Secondly, we exploit the probably little-known potential of multilevel models to identify the regional and country-level characteristics and/or public policies associated with resilient behaviour (via random intercept models) and examine the impact of a shock such as COVID-19 on regional vulnerability (via the random or stochastic part of the models).

The rest of this paper is structured as follows. ‘‘Conceptual Framework’’ section focuses on studying the conceptual framework of the 2021–2027 EU Cohesion Policy and the socio-economic vulnerability, the first step in the construction of a composite indicator, which in turn provides the basis for the selection and aggregation of single indicators. ‘‘Data and Variables’’ section  presents and justifies the dataset and single indicators used both to build the composite index and to develop the multilevel models. ‘‘Empirical Strategy ’ section describes the empirical strategy to build the composite index SEVI, as well as the multilevel modelling approach to study the shock that COVID-19 could represent. ‘‘Results’’ section  presents the main results of our analysis and examines some implications for public policies. Lastly, conclusions are drawn in ‘‘Conclusion and Discussion’’ section.

## Conceptual Framework

### European Union Cohesion Policy, 2021–2027

The five objectives of the Cohesion Policy for the period 2021–2027 are framed in the political guidelines for a strategic long-term vision to achieve the transition towards a green, digital and fair Europe. To do this, the EU must continue to develop as a social market economy, as outlined in the Europe 2020 Strategy (European Commission, 2020b). The social market economy is an integrated social, economic and political order characterised by having a market economic policy and a social policy. In turn, the social policy regulates the market economic policy. The latter is configured as its greatest difference from neoliberalism (European Commission, 2010).

Specifically, the five policy objectives drive investments to foster (European Commission, 2018):

1. A Smarter Europe through innovation, digitisation, economic transformation and support to small and medium-sized businesses.
2. A Greener, carbon free Europe, implementing the Paris Agreement and investing in energy transition, renewables and the fight against climate change.
3. A more Connected Europe, with strategic transport and digital networks.
4. A more Social Europe, delivering on the European Pillar of Social Rights and supporting quality employment, education, skills, social inclusion and equal access to healthcare.
5. A Europe closer to citizens, by supporting locally-led development strategies and sustainable urban development across the EU.

The underlying assumption is that these five priorities are mutually reinforcing: to improve education levels and increase investment in R&D, innovation and digitisation will improve competitiveness and economic growth in a sustainable way, thereby fostering job creation and reducing social exclusion. As is customary in the EU Cohesion Policy, the objectives of economic growth and job creation carry great weight, probably on the erroneous basis that social cohesion will follow from them (Sánchez & Ruiz-Martos, 2018). Accordingly, in the 2014–2020 period, the financial weight of the allocation criteria of the Structural Funds was 86% for relative wealth (per capita GDP) and 14% for labour market, education and demographic factors. However, a qualitative change was introduced for the period 2021–2027. In addition to the above criteria, youth unemployment, migration and greenhouse gas emissions will also be considered for the first time in the distribution of Structural Funds. More specifically, per capita GDP accounts for 81% of regional allocations; 15% of labour market, education and demographics allocations; 3% of migration allocations and 1% of climate change allocations (European Court of Auditors, 2019). The five goals are reviewed below.

Goals 1 and 3 are a continuity of previous planning periods, especially since the 2000–2006 period, when emphasis was placed on investment in R&D (Romer, 1994), human capital (Lucas, 1993), industrial innovation (Grossman & Helpman, 1994) and the provision of infrastructure or public capital (Aschauer, 1989) as drivers of economic growth. These models, inspired by the EU Cohesion Policy, integrated endogenous growth theory and argued that investment in these special categories of capital increased the productivity of all factors and therefore promoted economic growth. Subsequently, the concept of infrastructure was extended to research and innovation. Thus, the Horizon 2020 programme (financial instrument of the Europe 2020 Strategy to develop EU innovation policy since 2014) introduced the concept of research infrastructure (European Commission, 2011). Research Infrastructures are facilities that provide resources and services for research communities to conduct research and foster innovation. This concept aims to integrate research and innovation to promote market-related activities, which leads to a direct economic stimulus (European Commission, 2020c).

Goal 2 focuses on sustainable growth, which was introduced in the Europe 2020 Strategy as one of the pillars of the EU. Compared to other strategies, such as the Lisbon Strategy, Europe 2020 constituted a step forward. Since the publication of the *Brundtland Report* in 1982, there has been growing awareness of the importance of achieving a balance between the economic, social and environmental subsystems. Sustainable economic growth is understood as a growth rate that can be maintained without creating other significant problems, such as the depletion of resources or environmental problems, especially for future generations. This goal is rooted in the EU’s objective of competitive sustainability and cohesion through a new growth strategy: the European Green Deal. The key aim is to shift towards a sustainable and inclusive economic model, enabled by a broader diffusion and uptake of digital and clean technologies (European Commission, 2021a).

Due to the negative effects of the economic crisis on certain groups (the senior, youth, women, migrants and lower-skilled workers), goal 4 of the Cohesion Policy focuses on fostering inclusive growth by promoting the European Pillar of Social Rights. In turn, in 2021, and given that the effects of COVID-19 affected these groups more, a new ‘social rulebook’ has been introduced in the European Pillar of Social Rights to enhance social rights and strengthen the European social dimension across all policies of the Union (European Commission, 2021c). The main lines of action that should guide policy decisions in the Member States and their regions, including the programming of the 2021–2027 Cohesion Policy and the national recovery and resilience plans (European Commission, 2021c, p. 10), are aimed at reducing the gender employment gap, decreasing the rate of youth unemployment, reducing early school leaving and fostering higher education. The underlying idea is that special attention needs to be paid to young people and the low skilled (including migrants in both categories), who are more vulnerable to labour market fluctuations. Likewise, the demographic trends of the EU, marked by an ageing society, represent challenges for the principles of the Pillar of Social Rights, which focus on promoting health and care and ensuring that everyone in old age has the right to resources that ensure living with dignity.

Finally, goal 5 aims at promoting locally-led development strategies and sustainable urban development, with the objective of satisfying local objectives and needs and contributing to the smart, sustainable and inclusive growth of the EU. This local development strategy has also been a key factor in the EU Cohesion Policy since the 2000–2006 period. The approach is largely inspired by local development theories whose basic idea is to identify and enhance competitiveness factors at the local level (see Scott & Garofoli, 2007). COVID-19 has highlighted the importance of strengthening the resilience of urban areas to promote the well-being of inhabitants with challenges such as sustainable mobility and consumption, the treatment of urban waste through recycling, or the need for housing for new urban dwellers (European Commission, 2020b).

### Regional Socio-Economic Vulnerability in the European Union

Studies on vulnerability have been carried out in a range of fields. The fields that have probably received the most attention are poverty (Acconcia et al., 2020; Azeem et al., 2016; Gallardo, 2020), climate change, and physical vulnerability to natural disaster (Halkos et al., 2020; Marulanda Fraume et al., 2020) and financial or economic vulnerability (Alessi et al., 2020). Vulnerability is defined in a various ways in the literature (for a review, see Acconcia et al., 2020; Gallardo, 2018; Mina & Imai, 2016), so a crucial step of this study is to define the conceptual framework of socio-economic vulnerability. This is also important because the conceptual framework will determine the empirical strategy of our study.

**Fig. 2:**
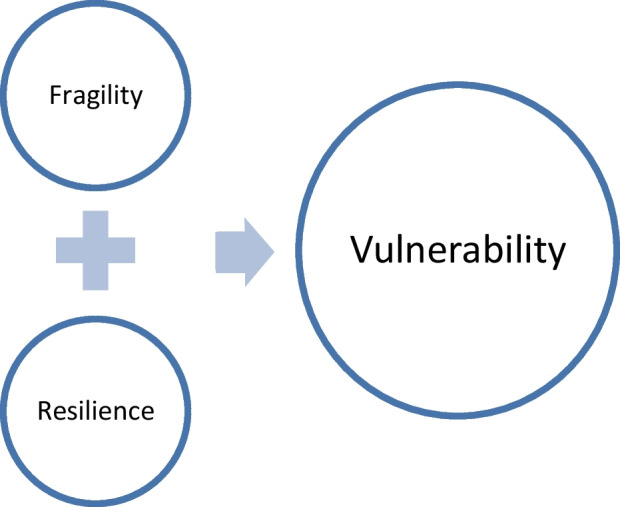
Components of socio-economic vulnerability

In general terms, vulnerability refers to the propensity or predisposition to be adversely affected together with the difficulty of reacting. The most recent vulnerability studies encompass a variety of concepts grouped into two broad forms: sensitivity or fragility to suffer harm, and the capacity to cope and adapt or resilience (Azeem et al., 2016; Halkos et al., 2020; Marulanda Fraume et al., 2020). Figure 2 shows this idea.

Under this framework, socio-economic fragility refers to the predisposition to suffer harm from the disadvantageous conditions and relative weaknesses related to social and economic factors (Cardona, 2004). In this vein, the *2020 Strategic Foresight Report* (European Commission, 2020b) identifies groups and areas that have suffered the effects of the pandemic most and face greater difficulties in coping with the effects of the COVID-19 shock. For example, residential care facilities and support services for older people and persons with disabilities were structurally fragile and unprepared to cope with and control the spread of the coronavirus. Other groups that have shown to be more fragile are students from disadvantaged backgrounds because they were less likely to benefit from online learning and lower skilled workers that were more likely to be employed in ‘contact jobs’ with greater exposure to the virus.

On the other hand, resilience is the ability to face shocks and persistent structural changes (e.g. digital transformation, globalisation and climate change) that affect people and society in such a way that current societal well-being or quality of life is preserved (Alessi et al., 2020; Benczur et al., 2020). Therefore, a resilient society aims to sustain its level of individual and societal well-being in an intergenerational fair distribution, that is, by ensuring current well-being without seriously compromising that of future generations (Manca et al., 2017, p. 6). Adaptation and transformation are key to bouncing forward. In this regard, the *2020 Strategic Foresight Report* (European Commission, 2020b) highlights that the EU’s social and economic resilience rests on its population and its unique social market economy. Among the key points to enhance resilience against COVID-19 are access to education and social protection, flexible work arrangements and a highly skilled workforce. Consequently, in the context of the EU 2021–2027 Cohesion Policy, the degree of a region’s socio-economic vulnerability might be estimated by a composite indicator built from a system of single indicators able to take into account these policy goals. At the same time, this system of indicators should allow identifying the socio-economic weaknesses of the regions, as well as defining the social and economical dimensions related to how a region is able to respond to the pressure from these dimensions, and whether it is capable of adapting to those pressures to deliver well-being in a sustainable way. Under this framework, the situation of a region with a greater degree of socio-economic vulnerability might be understood as having greater obstacles or found in a worse position to achieve the Cohesion Policy goals (2021–2027). In short, our premise is that socio-economic vulnerability is a latent variable, since it is a concept or construct which cannot be measured or estimated directly, but rather indirectly using collectable social and economic indicators.

Once we achieve an instrument to analyse the socio-economic vulnerability of EU regions in terms of the 2021–2027 Cohesion Policy goals, the next step is to study how a situation of economic and social stress such as the COVID-19 pandemic could affect regional vulnerability. In this vein, societies that are more resilient to disturbances will also be able to ensure a higher level of well-being or quality of life as the shock will have a less severe impact on them (Alessi et al., 2020; Manca et al., 2017). Taking into account the magnitude and duration of the COVID-19 effects, especially compared to previous experiences such as the SARS outbreak of 2003 (see for instance Lee & McKibbin, 2004; Keogh-Brown & Smith, 2008), it is reasonable to hypothesise that regions’ socio-economic vulnerability will not only be affected by their particular variables or characteristics, but also by the country’s characteristics (for example, public policies at the country level). This region-country interrelation may determine the degree to which a region is affected by the COVID-19 shock. Econometric multilevel modelling is a proper quantitative method to address these issues since it allows incorporating observed variables at both the regional and country levels among the explanatory variables.

## Data and Variables

### Data and Single Indicators to Build the SEVI

To develop the socio-economic vulnerability index (SEVI) in the EU regions, we use the official statistics of EUROSTAT and OECD at the NUTS-2 level which is the basic unit for the application of regional policies. We work with the most recent regional territorial classification, known as NUTS 2016, which entered into force on 1 January 2018 in accordance with the Commission Regulation (EU) 2016/2066. The overseas NUTS-2 territories have not been taken into account in this study (Ceuta and Melilla in Spain; and Guadeloupe, Martinique, Guyane, La Réunion and Mayotte in France). A total of 233 EU regions or NUTS-2 territories are studied.

In order to develop a system of indicators capable of representing how a region is able to respond to the pressures and challenges of the 2021–2027 Cohesion Policy, we selected 16 single indicators. For the system of indicators to be balanced, eight representative indicators of the socio-economic weakness or fragility of the regions and eight representative indicators of the capacity of the regions to face challenges or structural changes have been chosen. The eight single indicators of fragility have positive polarity, which means that an increase in the indicator could also lead to an increase in socio-economic vulnerability. Conversely, the eight single indicators of resilience have negative polarity, which means that an increase in the indicator could lead to a reduction in vulnerability. Appendix Table 5 presents the definitions and technical information of the single indicators. The values of the single indicators have been obtained as the average of the last two available years, including in all cases (except R&D) the year 2017, as is the usual practice in matters of EU Cohesion Policy.

The selection of single indicators has essentially been guided by the five goals set by the European Commission (2018) for the Cohesion Policy 2021–2027 mentioned above, as well as by plans, strategies and projects of the EU approved in the context of the COVID-19 crisis that also use monitoring indicators in areas related to the five goals of the EU Cohesion Policy. Appendix Table 6 displays the 16 single indicators with the EU official documents that guided our choice of single indicators indicated in the right column. In any case, our selection has been determined by the availability of statistical information, which is quite scarce at the NUTS-2 level in several areas such as climate change, income inequality and self-reported measures. Table 1 shows the descriptive statistics of the single indicators.

**Table 1:** Descriptive statistics of socio-economic vulnerability indicators for the EU27 regions in 2016–2017 (N = 233 NUTS-2)

	Mean	SD	Min	Max	CV	Region baseline
Early leavers	10.21	4.87	1.35	27.35	47.72	HR03—Jadranska Hrvatska
PM2.5	12.89	4.27	4.40	28.28	33.16	PT20—Região Autónoma Açores
Senior people	9.48	2.11	4.57	15.52	22.28	NL23—Flevoland
Male unemployment	7.94	4.80	1.85	24.15	60.52	CZ01—Praha
Female unemployment	8.78	6.95	1.90	39.25	79.22	DE22—Niederbayern
Youth unemployment	20.39	12.89	3.60	57.15	63.21	DE93—Lüneburg
Migrant	3.23	4.36	0	38.80	134.91	Regions with negative rate
Assault & crime	0.74	0.52	0.08	4.24	70.61	FRC2—Franche-Comté
R&D business	0.98	0.96	0	8.06	97.31	DE91—Braunschweig
R&D state	0.61	0.44	0	2.52	71.31	DEB2—Trier
Tertiary education	29.12	9.00	11.80	55.00	30.89	PL91—Warszawski stoleczny
Human resources in technology	31.92	8.38	13.95	54.70	26.24	PL91—Warszawski stoleczny
Registered community designs	3,591.21	4,000.26	0	24,813.07	111.39	ITH4—Friuli-Venezia Giulia
Internet	97.33	2.69	87.50	100.00	2.77	Several regions with 100
E-Administration	51.42	20.09	4.50	92.00	39.08	DK01—Hovedstaden
GDP-Gini	19,782.30	7,905.24	5,459.60	52,512.45	39.96	LU00—Luxembourg

HR is Croatia, PT Portugal, NL Netherlands, CZ Czech Republic, DE Germany, FR France, PL Poland, IT Italy, DK Denmark, LU Luxemburg

Next, we discuss the rationale that justifies the relationship between each single indicator and the composite indicator of socio-economic vulnerability (that is, the polarity). We start with the indicators of fragility and then illustrate why they might be considered indicators of fragility based on the literature and EU official documents and reports.

Dropping out of school has negative effects both for individuals and society (unemployment, less lifetime earning, more risk of poverty, higher public spending for social protection, etc.), hence the reduction of the percentage of people who dropped out of primary and secondary studies until a maximum of 10% is a target set out in the European 2020 Strategy in order to attain social cohesion in the EU (European Commission, 2010). The new ‘social rulebook’ of the European Pillar of Social Rights (European Commission, 2021c) identifies the reduction of early school leaving as one of the priorities of the 2021–2027 Cohesion Policy to foster inclusive growth. In the same vein, the prototype dashboard for social and economic resilience (European Commission, 2020b) considers early school leavers as a factor of socio-economic vulnerability in the category of social distress.

Inhaling PM2.5 has negative effects for health, among them respiratory and cardiovascular morbidity and lung cancer (World Health Organization, 2013). Moreover, this higher incidence of illnesses also puts greater pressure on public finances through health programmes and social benefits (sick leave, for example). PM2.5 air pollution is considered an indicator of vulnerability in the prototype dashboards for the geopolitical, green and digital dimensions of resilience because it constitutes an environmental threat (European Commission, 2020b).

Overall, older people who have left the labour market have lower average incomes and are more exposed to poverty than the rest of population (Marical et al., 2008; Peichl et al., 2012). Due to the uncertainty caused by the pandemic, even lower birth rates are expected in the EU and greater population ageing. Because people older than 65 constitute a healthcare burden and are at greater risk of poverty, they are considered a factor of vulnerability in the prototype dashboard for social and economic resilience (European Commission, 2020b). The European Pillar of Social Rights Action Plan also considers that special attention needs to be devoted to older people to promote health and care and ensure they live in dignity (European Commission, 2021c).

Higher levels of unemployment offset economic development processes since these are linked with lower standards of living and social problems (for example, robberies, crimes, etc.), so that unemployment reduces life satisfaction of the wider population (Chadi, 2014; Helliwell & Huang, 2014). In addition, during the COVID-19 pandemic, people not working for involuntary reasons were at greater risk of suffering mental disorders (Yao & Wu, 2021) and more likely to self-report more physical and mental health problems (Ikeda et al., 2021). It is also convenient to include women’s unemployment and youth unemployment because in the EU27 they reached values above men’s unemployment in 2019 (6.9%, 15.3% and 6.3% respectively, Eurostat information), and because they add specific aspects of fragility to the regions. Female unemployment is one of the social conditions most strongly correlated with income inequality (Kollmeyer, 2013; Sánchez & Pérez-Corral, 2018) and is an explanatory factor for the higher incidence of risk of poverty in older women than in older men (Dessimirova & Bustamante, 2019). For its part, youth unemployment contributes to deteriorating their resilience, optimism, autonomy and overall life satisfaction (Merino et al., 2019). Unemployment rate is considered an indicator of economic vulnerability in the prototype dashboard for social and economic resilience (European Commission, 2020b). The European Pillar of Social Rights Plan distinguishes unemployment rates by groups of people and indicates, among its objectives, the reduction of the gap in male and female employment rates (European Commission, 2021c). It also defends that special efforts need to be devoted to young people who are more vulnerable to labour market fluctuations.

Migrants are likely to be one of the most vulnerable population groups, whether displacement is due to economic reasons or forced by violence. Migration has negative effects on quality of life because people’s family and social ties break down and they are more exposed to poverty (Sánchez Mójica, 2013). Within the context of the COVID-19, migrant workers in the EU are very vulnerable because they are more likely to be in temporary employment, earn lower wages and have jobs that are less amenable to teleworking (Fasani & Mazza 2020). In the same vein, the European Pillar of Social Rights Action Plan states that the 2021–2027 Cohesion Policy should pay special attention to migrants since they are more vulnerable to fluctuations in the labour market (European Commission, 2021c). Additionally, as we indicated in a previous section, for the first time, migration will receive 3% of the Structural Funds in the 2021–2027 Cohesion Policy (European Court of Auditors, 2019). Under this approach, positive migration ratios are considered a factor of socio-economic fragility and a greater pressure on public finances, and in those regions where the migration ratio is negative its values have been replaced by zero.

A prevalence of assaults and criminal activities creates unstable environments and deters investment in productive activities, is negatively related to quality of life and slows down sustainable urban development (Chica-Olmo et al., 2020). As a consequence of COVID-19 economic hardships have worsened, so this situation may also lead to higher exposure to organised crime and a rise in corruption (European Commission, 2020b, pp. 10–11). Crime and assault rates are a factor of fragility that increase socio-economic vulnerability directly in the cities or towns where they are registered.

We now examine why the rest of the indicators are considered indicators of resilience. Gramillano et al. (2018) analysed the indicators most frequently used by the Directorate-General for Regional and Urban Policy of the EU to assess the effectiveness in achieving the innovation and digitalization priorities of the previous EU Cohesion Policy period (2014–2020). They concluded that private investment in research and innovation, as well as enterprises receiving support from research institutions, can measure the networking activity and be proxies for potential technological transfer and knowledge exchange. As widely used indicators of innovation, the authors highlight the number of enterprises that introduce new services, products or processes. The five indicators of innovation and digitisation of our system are considered as proxies of a region’s intellectual assets in the Regional Innovation Scoreboard for 2021 developed by the European Commission to assess innovation performance, namely the relative strengths and weaknesses of European regions (European Commission, 2021b). The idea is that innovation and a highly educated and well-trained workforce are critical to the development of a competitive, smart and knowledge economy. Education and innovation capacity, including product creativity and design – as a link between innovation and the market – are key factors in determining the recovery of regions before a shock (economic crises, for example). In this vein, the European 2020 Strategy set targets for Members States in terms of R&D investment (3% of GDP) and tertiary educational attainment (minimum 40% of the population aged 30–34) (European Commission, 2010). Expenditure on R&D, both private and public, is considered an indicator of economic growth and innovation that fosters socio-economic resilience in the prototype dashboard for social and economic resilience (European Commission, 2020b). Likewise, registered community designs per billion GDP is one of the outcome indicators of goal 1 of the 2021–2027 Cohesion Policy (European Commission, 2018).

The use of the Internet is an increasingly crucial factor for competitiveness and economic security, as it determines the capacity of territories to compete in and benefit from the knowledge-based economy. Studies with a territorial approach have shown that the availability of high-speed networks is a key determinant of quality of life because it facilitates economic, educational and social connections (Sánchez et al., 2018). On the contrary, the lack of Internet could represent a digital divide that increases levels of economic and social inequality. The COVID-19 crisis underscored the importance of households having internet access. During the lockdown, people relied more on online communication via the Internet for attending schools, buying daily necessities and working from home (Shek, 2021). Thus, the prototype dashboards for the geopolitical, green and digital dimensions include digital skills, teleworking capacity and e-health among the capacity indicators of digital resilience (European Commission, 2020b). Additionally, the percentage of individuals who use the Internet for interactions with public authorities or the e-Administration is considered an indicator of digital capacity that fosters regional resilience in the prototype dashboards for these same dimensions (European Commission, 2020b).

Per capita GDP is the main indicator considered by the European Commission (2018) for the allocation of the Structural Funds because it is the most neutral measure and reliable indicator and reflects the needs and disparities of the regions and Member States (European Court of Auditors, 2019). Under the scope of resilience, the ability to save is key for helping families and companies cope with adverse situations (Alessi et al., 2020; Benczur et al., 2020; European Commission, 2020b; Le Blanc, 2020). Taking into account the negative social and economic effects of income inequality (for a review, see Sánchez & Pérez-Corral, 2018; Sánchez & Ruiz-Martos, 2018), we consider the regional indicator proposed by Sen (1976), that is, GDP adjusted by the Gini index of each country (the Gini for NUTS-2 is not available).

### Variables for the Multilevel Modelling

The explanatory variables of the multilevel models come from level 1 or region and level 2 or country. More specifically, we consider monetary poverty at regional level, and government expenditure in education and political stability at country level (see Appendix Table 7). According to the conceptual framework of this study, the choice of these three variables has been guided by the assumption that socio-economic vulnerability can be induced and/or explained by the sensitivity or fragility to harm and adaptive capacity or resilience. Several works have studied resilience and the impact of COVID-19 in the EU and conclude that one of the main ways to deal with a shock such as falling income is to use one’s own savings (Alessi et al., 2020; Giovannini et al., 2020; Le Blanc, 2020; Manca et al., 2017). That is, family savings can act as financial buffers for households in the wake of the COVID-19 crisis. In addition, these works highlight that being at the bottom of the income distribution and/or living in a poor neighbourhood increases the chances of not knowing how to cope with a situation of distress. In this vein, the percentage of people in a region with an income below 60% of the region's median income (variable *Poverty* in our models) could be a proxy for the degree to which a region would be adversely affected by the pressure of the pandemic, as well as the capacity to deal with the shock.

The two country-level variables we have chosen (*Education* and *Stability*) aim to account for the role of the public sector in regional vulnerability (SEVI). The literature referred to in the previous section highlights the importance of human and social capital as drivers of resilient behaviour and an adaptive capacity to deal with shocks. Thus, government expenditure in education as a merit good that fosters citizen participation (more democratic societies), equal opportunities and lower income inequality (Sánchez & Pérez-Corral, 2018) could favour a society’s adaptation capacity, as well as promote the opportunity to bounce forward. Lastly, in situations of market economy stress, political stability and good governance ensuring compliance with contracts are essential to guarantee the functioning of the markets (Chang, 2011). Likewise, in a crisis context such as the COVID-19 pandemic, people can be more resilient when they trust in the institutions and live in a society that provides a safe and prosperous environment (Bittmann, 2021; Giovannini et al., 2020). Table 2 shows the descriptive statistics of the variables.

**Table 2:** Descriptive statistics of socio-economic vulnerability index (SEVI) and variables of multilevel modelling EU27, 2018

	Mean	SD	Min	Max	CV	Sample size
SEVI	1.25	0.23	0.66	1.74	18.43	233 NUTS-2
Poverty	16.44	5.83	4.10	41.40	35.44	233 NUTS-2
Education	4.84	0.96	3.00	6.80	19.94	27 Member States
Stability	0.69	0.36	0.06	1.37	51.32	27 Member States

## Empirical Strategy

### Building the SEVI

The choice of mathematical method for aggregating the single indicators into a composite indicator will depend on the kind of measurement model that best fits the phenomenon being analysed (Maggino, 2017). The conceptual framework to analyse the socio-economic vulnerability of EU regions, provided in a previous section, led us to develop our model under the scope of a formative model. In formative measurement models, causality flows from the single indicators to the latent variable, since single indicators are viewed as causes of the latent variable (see Diamantopoulos et al., 2008; Jiménez-Fernández & Ruiz-Martos, 2020). For instance, in our case, the socio-economic vulnerability index (SEVI) of a region includes indicators of innovation, education, unemployment, pollution, etc. Any change in one or more of these components (even if the other factors do not change) is likely to cause a change in a region’s SEVI score (the latent construct). However, if a region’s SEVI decreases, it would not necessarily be accompanied by an improvement in all of the components (single indicators).

Keeping this in mind, we applied an iterative distance methodology based on the Distance P2 introduced by Pena Trapero (1977) and applied in several works (see Cuenca-García et al., 2019; Sánchez et al., 2018; Sánchez & Ruiz-Martos, 2018; Zarzosa Espina & Somarriba Arechavala, 2013). We use the metric structure in the R^m^ vector space, where *m* is the number of single indicators. This allows us to obtain a composite indicator that measures distances to perform benchmarking between the units studied in order to develop the socio-economic vulnerability indicator (SEVI) of the 233 European regions or NUTS-2.

In our case, the composite indicator represents a weighted Euclidean metric that is defined as follows (see Jiménez-Fernández et al., 2022):

1 $${\mathrm{SEVI}}_{\mathrm{i}}={(\sum_{\mathrm{j}=1}^{\mathrm{m}}{\left|{\mathrm{x}}_{\mathrm{ij}}-{\mathrm{x}}_{*\mathrm{j}}\right|}^{2}{\mathrm{w}}_{\mathrm{j}})}^{1/2}$$

where *m* is the number of single indicators, *x*_*ij*_ is the value of the *j*-th indicator in the *i*-th region, x_*j_ is the *j*-th value in the reference vector X_*_ = (x_*1_, x_*2_,…,x_*m_) and *w*_*j*_ is the weight of the *j*-th single indicator.

Given that the single indicators often have different measurement units, the single indicators {*x*_*1*_*,…,x*_*j*_} have been normalised using Min–Max normalisation in order to make them comparable.

Our method considers in its calculation formula the distance between each individual indicator and the most desirable situation taken from the reference vector. The reference vector (X_*_) is like a hypothetical region that, in the set of all EU regions, registers the best values of all single indicators. Thus, we take into account the complete empirical distribution in the 233 EU regions. More specifically, for single indicators with positive polarity, we select the minimum value of the indicator in the entire sample. For instance, early leavers is a single indicator with positive polarity: the higher the early leavers rate is in a region, the greater the region’s vulnerability. The hypothetical best region (the least vulnerable) will register the lowest rate of early leavers, that is, the minimum value of all the regions. For single indicators with negative polarity, the reference value is the maximum value of the sample. For example, for R&D investment, the higher the value is in a region, the less vulnerable it is. In this case, the hypothetic best region or the least vulnerable will register the maximum value in R&D investment. Proceeding in this way, the SEVI composite indicator will take higher values, the greater the distance it is with respect to the most desirable values of the individual indicators. That is, the greater the SEVI, the more vulnerable or the worse the performance of a region in the different indicators studied. Consequently, we can quantify and compare all the regions under analysis.

The weights of the single indicators (*w*_*j*_) are computed using unsupervised machine learning algorithms. More specifically, we use multivariate adaptative regression splines (MARS) to identify the best functional relationships between the composite indicator and the set of single indicators. In this way, *w*_*i*_denotes the importance of each indicator according to its contribution to the SEVI and avoids potential multicollinearity issues. For a more detailed approach to this methodology and its mathematical properties, see Jiménez-Fernández et al. (2022).

### The Impacts of Shocks on Socio-Economic Vulnerability of EU regions

The second aim of this paper is to check whether country characteristics interact with regions’ characteristics to alter patterns of socio-economic vulnerability. In other words, we consider the possibility that two regions randomly selected from the same country will register a more similar level of socio-economic vulnerability than two regions randomly selected from different countries. This would mean that we assume no independence among regions belonging to the same country. To test this hypothesis, multilevel models should be used. In a classical one-level model it is assumed that the observations are independent, and the error is treated as noise, so the estimate should minimise the error. However, when the data is nested, the correlation between observations within a group could be different from the correlation between groups, resulting in two types of errors. An advantage of multilevel models is that they analyse what part of the random error is due to the effect of level 2 (country) and what part is due to level 1 (regions) (see Goldstein, 2011; Snijders & Bosker, 2012). That is, multilevel modelling allows us to determine what part of the variability in the regions’ socio-economic vulnerability can be explained by country characteristics.

Likewise, multilevel modelling distinguishes between the fixed or deterministic part of the model and the random or stochastic part, thus enabling a two-directional analysis. Firstly, by estimating the signs and values of the model parameters (fixed part of the model), we can study how changes at the regional (level 1) and country level (level 2) influence SEVI, as well as identify the regional and country-level characteristics associated with resilient behaviour. Secondly, the random part of the models could inform us on how a shock, such as COVID-19, would impact on regional vulnerability (third objective of this paper). In turn, in the random part of the model, we can analyse the possible idiosyncratic and covariate shocks caused by COVID-19. That is to say, we can identify what proportion of the variability in vulnerability (SEVI) not explained by the model (stochastic or random effects) is attributable to regional-level characteristics (idiosyncratic effects) or to the interaction between the country characteristics and the regional characteristics (covariate effects).

Next, we present two different specifications to estimate multilevel models which will allow us to check the aims or hypotheses 2 and 3 of this study.

#### Specification 1: Multilevel Random Intercept Model

We consider a two-level structure where regions *i* (level 1) are nested or hierarchised into countries *j* (level 2). The random intercept model accounts for country differences in SEVI. In this specification, the intercept varies randomly between the countries, but the slope is the same for all of them. Let *SEVI*_*ij*_ be the value of the socio-economic vulnerability index in region *i* and country *j*, where *i* Є {1,…, 233} and *j* Є {1,…,27}. For each observation located in the *j*-country, the model can be written as follows:

2 $${\mathrm{SEVI}}_{\mathrm{i}}={\upbeta }_{0\mathrm{j}}{\upbeta }_{1}{\mathrm{x}}_{\mathrm{ij}}+{\mathrm{e}}_{1\mathrm{j}}$$

where ${\beta }_{0j}{=\beta }_{0}{u}_{j}$, ${x}_{ij}$ Є X, being X a nxm-dimensions matrix of observed explanatory variables both at regional and country level, and *β*_*1*_ its associated parameters. For country *j*, the intercept is *β*_*0j*_, which may be smaller or larger than the intercept of population *β*_*0*_. The country random effects are denoted by *u*_*j*_ and the regional residuals (with nxm dimensions) are denoted by *e*_*ij*_. The residuals *u*_*j*_ are assumed to have a normal distribution of zero mean and variance${\sigma }_{u}^{2}$. In order to identify the fixed and random parts of the model, Eq. (2) can be written as:

3 $${\mathrm{SEVI}}_{\mathrm{ij}}={\upbeta }_{0}+{\upbeta }_{1}{\mathrm{x}}_{\mathrm{ij}}+{\mathrm{u}}_{\mathrm{j}}+{\mathrm{e}}_{\mathrm{ij}}$$

In this equation, the fixed part of the model shows the relationship between the mean of *SEVI* and the explanatory variables (*β*_*0*_ + *β*_*1*_*x*_*ij*_ with parameters *β*_*0*_*, β*_*1*_*),* and the random part captures the residuals from different levels (*u*_*j*_ + *e*_*ij*_ with variances ${\sigma }_{u}^{2},{\sigma }_{e}^{2}$).

Following this specification, we estimated the null model (without explanatory variables) and Model 1, which includes the variables *Poverty, Education* and *Stability*. The null model allows us to check if the structure of socio-economic vulnerability in the EU regions is nested, that is, whether there is an interaction between the regional-level and country-level variables (objective 2). If a nested structure is confirmed, multilevel modelling would be a suitable approach because one-level modelling could lead to incorrect conclusions (see Goldstein, 2011; Snijders & Bosker, 2012).

#### Specification 2: Random Slope Model for Poverty Variable

Specification 2 is an extension of the random intercept model which also considers that the slope for the variable *Poverty* varies randomly among the different countries. Let *SEVI*_*ij*_ be the variable that indicates the value of the socio-economic vulnerability index in region *i* of country *j*, where *i* Є {1,…, 233} and *j* Є {1,…,27}. For each observation located in *j*-country, the model can be written as follows:

4 $${\mathrm{SEVI}}_{\mathrm{ij}}={\upbeta }_{0\mathrm{j}}+{\upbeta }_{1\mathrm{j}}{\mathrm{p}}_{\mathrm{ij}}+{\upbeta }_{2}{\mathrm{x}}_{\mathrm{ij}}+{\mathrm{e}}_{\mathrm{ ij}}$$

where *β*_*0j*_ = *β*_*0*_ + *u*_*0j*_ and *β*_*1j*_ = *β*_*1*_ + *u*_*1j*_; *x*_*ij*_ Є X, being X a nxm-dimensions matrix of observed explanatory variables at both regional and country levels, and *β*_*2*_ its associated parameters. The variable *Poverty* is denoted by *p*. The average regression for *Poverty* has slope *β*_*1*_and the slope for each country is *β*_*1j*_. The random errors *u*_*0j*_ and *u*_*1j*_ are assumed to have a normal distribution of zero mean and variance ${\sigma }_{u0}^{2}$ and ${\sigma }_{u1}^{2}$, respectively. Model 2 is estimated following this specification. Developing Eq. 4, we can identify the fixed and random parts of the model:

5 $${\mathrm{SEVI}}_{\mathrm{ij}}={\upbeta }_{0}+{\upbeta }_{1}{\mathrm{p}}_{\mathrm{ij}}+{\upbeta }_{2}{\mathrm{x}}_{\mathrm{ij}}+{\mathrm{u}}_{0\mathrm{j}}+{\mathrm{p}}_{\mathrm{ij}}{\mathrm{u}}_{1\mathrm{j}}+{\mathrm{e}}_{\mathrm{ ij}}$$

In this Eq. (5), the fixed part of the model shows the relationship between the mean of *SEVI* and the explanatory variables (*β*_*0*_ + *β*_*1*_*p*_*ij*_ + *β*_*2*_*x*_*ij*_ with parameters *β*_*0*_*, β*_*1*_*, β*_*2*_*),* and the random part captures the residuals from different levels (*u*_*0j*_ + *c*_*ij*_*u*_*1j*_ + *e*_*ij*_ with the parameters ${\sigma }_{u0}^{2}$, ${\sigma }_{u1}^{2}$, ${\sigma }_{e}^{2}$; where *p*_*ij*_*u*_*1j*_ is the interaction between the country and *Poverty*).

Following this specifications, we estimate Model 2, where all the explanatory variables are included (*Poverty, Education* and *Stability*).

#### Idiosyncratic Effects and Covariate Effects

Changes in socio-economic vulnerability caused by a shock such as COVID-19 can be introduced and analysed throughout the random or stochastic part of the multilevel models (see, for instance, Halkos et al., 2020). In turn, within the random part, we can distinguish what proportion of the variability in vulnerability (SEVI) not explained by the model is attributable to regional-level (idiosyncratic effects) or country-level effects and the interrelation between country and regional levels (covariate effects). In this vein, the interclass correlation (ICC) informs what part of the random effects would be explained by covariate effects. That is, the ICC informs us how changes in the environment or covariates of the regions could affect their socio-economic vulnerability.

In specification 1 with random intercept, the ICC can be calculated as follows:

6 $$\mathrm{ICC}=\frac{{\sigma }_{u0}^{2}}{{\sigma }_{u0}^{2}+{\sigma }_{e}^{2}}$$

where ${\sigma }_{e}^{2}$ is the residual variance and ${\sigma }_{u0}^{2}$ the variance between groups (countries).

In specification 2 with random slope, the variance between groups depends on the value of the variable *Poverty* (p) in each region; hence the ICC takes different values in each region. This information is interesting since it allows us to obtain a map of EU regions that shows the intensity of the effects of COVID-19 on their socio-economic vulnerability. The formula for calculating the ICC can be expressed as follows:

7 $${\mathrm{ICC}}_{\mathrm{i}}=\frac{\mathrm{Var}\left({\mathrm{u}}_{\mathrm{oj}}+{\mathrm{p}}_{\mathrm{ij}}{\mathrm{u}}_{1\mathrm{j}}\right)}{\mathrm{Var}\left({\mathrm{u}}_{\mathrm{oj}}+{\mathrm{p}}_{\mathrm{ij}}{\mathrm{u}}_{1\mathrm{j}}\right)+{\upsigma }_{\mathrm{e}}^{2}}$$

where *p*_*ij*_*u*_*1j*_ is the interaction between country *j* and the variable *Poverty* (p) at regional level, and where

8 $$\mathrm{Var}\left({\mathrm{u}}_{\mathrm{oj}}+{\mathrm{p}}_{\mathrm{ij}}{\mathrm{u}}_{1\mathrm{j}}\right)=\mathrm{Var}\left({\mathrm{u}}_{\mathrm{oj}}\right)+{\mathrm{p}}_{\mathrm{ij}}^{2}\mathrm{Var}\left({\mathrm{u}}_{1\mathrm{j}}\right)+2{\mathrm{p}}_{\mathrm{ij}}\mathrm{Cov}\left({\mathrm{u}}_{\mathrm{oj}},{\mathrm{u}}_{1\mathrm{j}}\right)={\upsigma }_{\mathrm{u}0}^{2}+{\mathrm{p}}_{\mathrm{ij}}^{2}{\upsigma }_{\mathrm{u}1}^{2}+2{\mathrm{p}}_{\mathrm{ij}}{\upsigma }_{{\mathrm{u}}_{0\mathrm{j},}{\mathrm{u}}_{1\mathrm{j},}}$$

## Results

### The Socio-Economic Vulnerability of EU regions

Focusing on the descriptive statistics for the 16 indicators of socio-economic vulnerability we have analysed (Table 1), the values of Pearson's coefficient of variation indicate that the largest territorial differences arose in the objective of fostering an innovative and smart economic transformation (especially in the indicators registered community designs, R&D business and R&D state), as well as in social rights (especially in migrant and female unemployment). The last column of Table 1 shows the regions that rank highest in each single indicator, namely our reference vector to build the SEVI. In other words, from a socio-economic viewpoint, the best theoretical region in the EU (the least fragile and most resilient) should register the values of the last column. The further a region is from this hypothetical region, the greater its socio-economic vulnerability and therefore the greater the value of its SEVI. Overall, we observe that two regions of eastern European countries (Croatia and Poland) register the best positions in the three indicators of human capital (early leavers, tertiary education and human resources in technology). Four regions of Germany invest the most in innovation (business R&D and state R&D) and have the lowest unemployment rates for both women and youth.

**Fig. 3:**
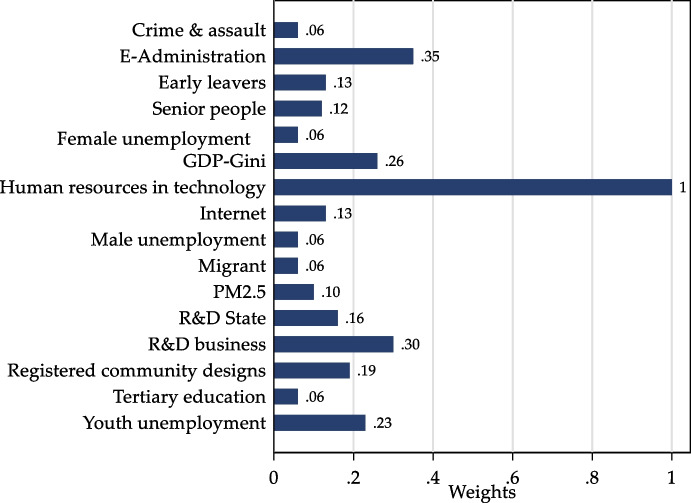
Weights of the single indicators of SEVI

The average value of the SEVI is 1.25. Hovedstaden in Denmark is the least socio-economically vulnerable region in the EU27 as it has the lowest SEVI (0.66), while Dytiki Makedonia in Greece is the most vulnerable (maximum SEVI value = 1.74) (Table 2). From a statistical viewpoint, SEVI is a variable that follows a normal distribution (Shapiro–Francia test, *z* = 1.552, *p* = 0.06027, *N* = 233; see Appendix Fig. 6). Figure 3 shows the weights assigned to each indicator; specifically, the proportion in which each indicator contributed to the metric and therefore to the SEVI. Resilience indicators, especially innovation and digitisation (goal 1 of the EU 2021–2027 Cohesion Policy), have the highest weights. Among the fragility indicators of the regions, youth unemployment, early leavers, senior people and PM2.5 register the largest weights.

**Fig. 4:**
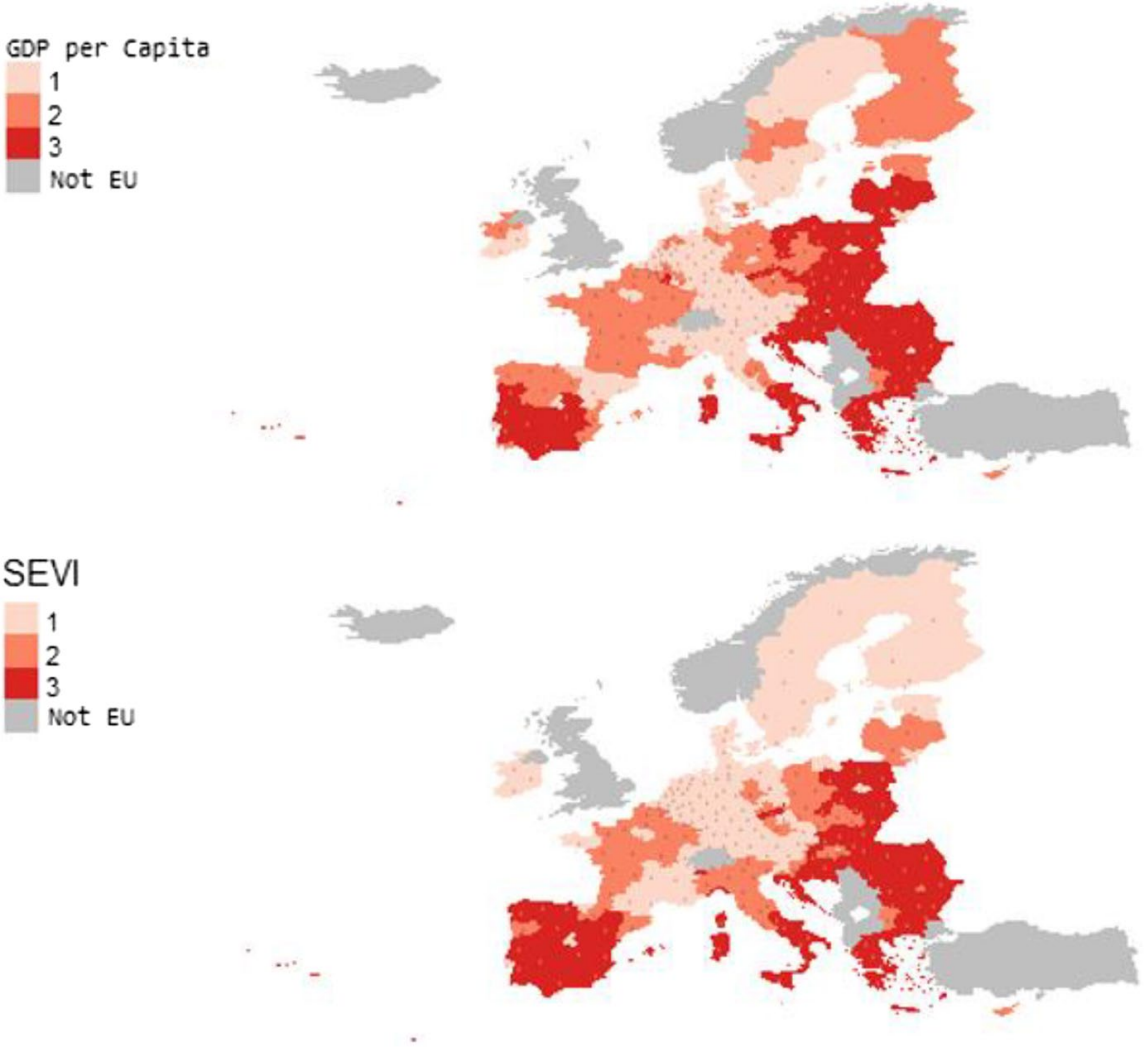
Classification of EU regions according GDP per capita and socio-economic vulnerability. *Note*. According GDP per capita: group 1 (47% of population residing in regions with GDP per capita above the GDP per capita of the whole of EU), group 2 (25% of population residing in regions with GDP per capita between 75 and 100% of the UE27), group 3 (28% of population residing in regions with GDP per capita below the 75% of the UE27). According the SEVI, regions are classified into three groups from less to higher socio-economic vulnerability with the next percentages of population: group 1 (46.35%), group 2 (26.40%) and group 3 (27.25%)

Following the European Commission’s proposal (2018) for the distribution of the Structural Funds, that is, taking as a reference the GDP per capita (average 2016–2017) and the population (average 2016–2018), the 233 NUTS-2 could be grouped into three blocks: 47% of the population of the EU27 would reside in regions where the GDP per capita is above the GDP per capita for the whole of the EU27, 25% of the population in regions with a GDP per capita between 75 and 100% of the EU27, and the remaining 28% of the population in regions where GDP per capita is less than 75% of the EU27. In order to analyse the implications for the 2021–2027 Cohesion Policy while maintaining the same budgetary effort, we take these population percentages as a reference to divide the EU regions into three groups according to the SEVI. Figure 4 displays the results of the SEVI grouped into the three types of regions analysed.

The regions that are in the most disadvantaged situation to face the challenges of the 2021–2027 Cohesion Policy, namely group 3 which represents 27.25% of the EU27 population are: all regions of Greece and Croatia; all regions of Romania, Bulgaria and Slovakia except the regions where their respective capitals are located; all regions of Hungary and Portugal except two; more than half of the territory of Spain and Poland; and the regions located in southern Italy. In contrast, the regions in the best position or group 1, which represent 46.35% of the EU27 population, are: Estonia and Malta; all regions of Denmark, Finland, Ireland, the Netherlands and Sweden; and all regions of Austria, Belgium and Germany except one. The rest of the regions (group 2) represent 26.40% of the EU27 population and are located mainly in France, northern Italy and the Czech Republic.

At a first glance, it might seem that the European Commission criterion for allocating the Structural Funds and our multidimensional proposal (SEVI) lead to similar results since the pairwise correlation between the GDP per capita and the SEVI for the whole set of 233 NUTS-2 is quite high (*r* = -0.77, *p* < 0.001). However, if we distinguish among the three groups of regions, the results are somewhat different. As Table 3 indicates, there is no correlation in the regions of group 2 between our proposal of socio-economic vulnerability and the one-dimensional criterion of the European Commission. Likewise, the correlation is low in group 3. Focusing on groups 2 and 3, we can identify which regions would be harmed in terms of the allocation of Structural Funds if the traditional criterion were applied. To do so, a single indicator can be taken as a reference of economic activity (GDP pc) instead of a set of indicators that complement the GDP and accurately reflect the socio-economic fragility and capabilities of the EU regions in order to face the challenges of the Cohesion Policy.

**Table 3:** Correlation between per capita GDP and SEVI by groups of EU regions

	Group 1	Group 2	Group 3
Correlation coefficient (r)	-0.6015	-0.1158	-0.3637
p-value	< .001	0.4280	0.0016
N	111	49	73
% population	46.35	26.40	27.25

The most remarkable outcome in groups 2 and 3 is that 12 out of 21 Italian regions, 11 out of 17 Spanish regions, two regions in Portugal, Corse in France, Attiki in Greece and Bucuresti-Ilfov in Romania would be negatively affected following the European Commission criterion. In other words, despite the fact that these regions surpass the thresholds of per capita GDP, according to our multidimensional criterion, they are more vulnerable and less resilient and should therefore attract more financial attention under the EU Cohesion Policy for the period 2021–2027. On the other hand, 12 regions located in Member States of the previous eastern Europe turn out to be less vulnerable from a socio-economic standpoint than their relatively low position in per capita GDP reflects.

### Multilevel Analysis: Socio-Economic Vulnerability and COVID-19

The results of the null model, the random intercept model (Model 1) and the random slope model (Model 2) are shown in Table 4. The results of the null model indicate differences in the socio-economic vulnerability of the regions across countries because the likelihood ratio (LR) test (X^2^(1) = 209.98, *p* < 0.001), which contrasts the multilevel model against the one-level OLS model, is significant. In fact, the value of the intraclass correlation (ICC = 0.70) might be interpreted as meaning that 70% of the variability in socio-economic vulnerability is attributable to differences across countries. Thus, the estimation of multilevel models that take into account the ‘country’ effect and the interaction between regional and country variables is justified.

**Table 4:** Multilevel modelling of the effects of regional and country characteristics on socio-economic vulnerability in the EU regions, 2017 (N_regions_ = 233; N_countries_ = 27)

	Null model	Model 1	Model 2
Fixed effects (p-value)			
Poverty (region level)		0.014 (< .001)	0.014 (< .001)
Education (country level)		-0.081 (0.002)	-0.075 (0.004)
Stability (country level)	1.232 (< .001)	-0.235 (0.001)	-0.255 (< .001)
Intercept		1.543 (< .001)	1.531 (< .001)
Random effects			
Variance intercept (${\upsigma }_{\mathrm{u}0}^{2}$)	0.03655	0.01337	0.00233
Variance poverty (${\upsigma }_{\mathrm{u}1}^{2}$)	–-	–-	0.00002
Covariance (u_0j_, u_1j_)	–-	–-	0.00021
95% conf. interval covariance	–-	–-	(0.00004–0.00038)
Variance residual (${\upsigma }_{\mathrm{e}}^{2}$)	0.01576	0.01032	0.01008
Intraclass correlation (ICC)	0.70	0.56	0.31–0.84(a)
Model fit			
-2Log Lik	-234.79	-346.91	-349.14
LR test, X^2^ (p-value)	209.98 (< .001)	157.09 (< .001)	159.31 (< .001)
R^2^m (fixed)	–-	54.32%	69.71%
R^2^c (fixed & random)	–-	80.01%	75.45%

(a) In the estimation with random intercept and random slope (Model 2), the variance (u_0_, u_1_) takes different scores for each value of the explanatory variable whose slope is considered to be random; thus the ICC yields different scores for each region

Models 2 and 3 incorporate the three explanatory variables *Poverty*, *Education* and *Stability*. Several goodness measures of the model are reported at the bottom of Table 4. In the framework of multilevel models, the marginal R-squared (R^2^m) represents the variance explained by fixed factors of the model and the conditional R-squared (R^2^c) represents the variance explained by fixed and random factors (see Nakagawa & Schielzeth, 2013). The difference between the corresponding R^2^c and R^2^m values reflects the amount of variability in the random effects. Both models 1 and 2 present high R^2^, the indicator -2 log likelihood decreases from model 1 to 2 and the result of the likelihood ratio (LR) test shows that Model 2 is an improvement over Model 1.

In both models (1 and 2), all the variables are statistically significant, and the signs of their estimated parameters are consistent with the literature. Namely, increases in regional monetary poverty would be associated with a rise in the socio-economic vulnerability of the regions, whereas increases in government expenditures in education and an improvement in self-reported political stability would lead to a reduction in vulnerability or foster the capacity for resilience. In addition, the results of Model 2, which analyses the relationship between vulnerability and monetary poverty for each country, indicate that increases in monetary poverty lead to greater socio-economic vulnerability in regions with a higher level of vulnerability (the covariance is positive and statistically significant).

**Fig. 5:**
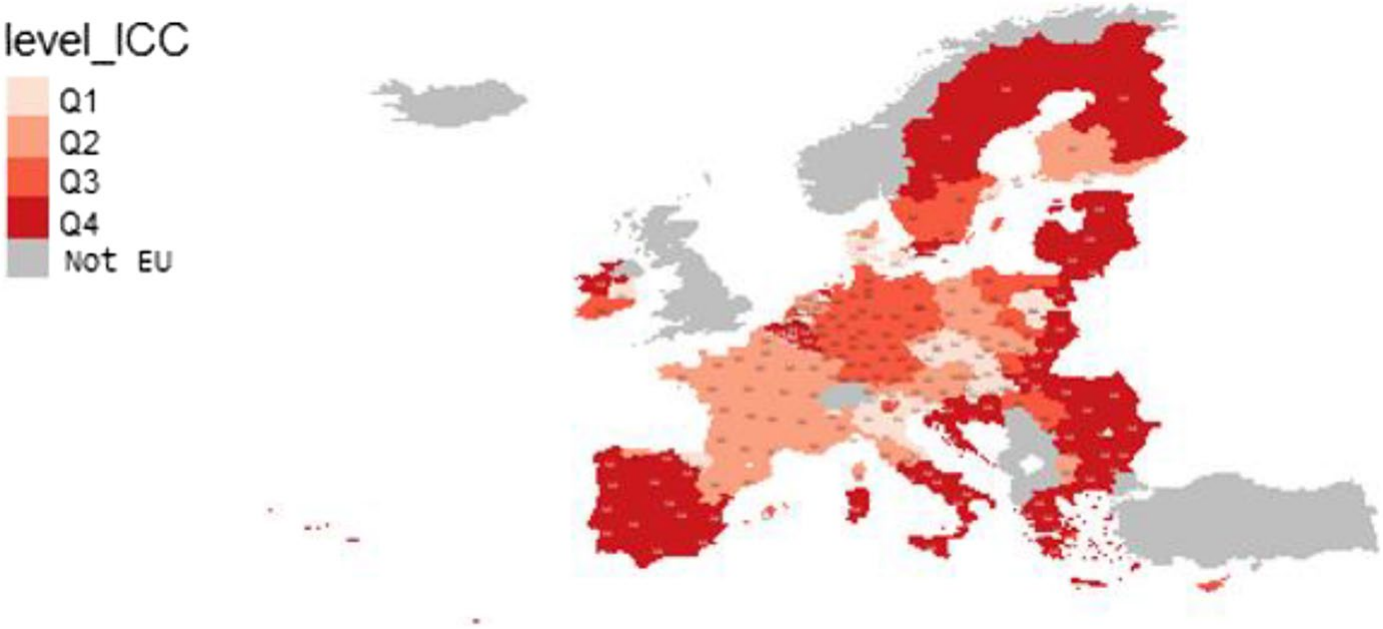
Covariate effects of COVID-19 on socio-economic vulnerability of EU regions. *Note*. ICC is intra-class correlation. Q1 (30.5%, 53.4%), Q2 (53.5%, 58.4%), Q3 (58.5%,61%), Q4 (61.1%,84.3%)

Focusing on the random or stochastic part of Model 2 and applying formulas (7) and (8), we calculated the ICC for each region. ICC provides the proportion of vulnerability variability not explained by the model that is attributable to changes in the environment or covariates of the regions, such as changes caused by the COVID-19 pandemic that could interact with each country’s characteristics and with each region’s poverty level. The ICC varies from 30.5% in Bucuresti-Ilfov (Romania) to 84.3% in Campania (Italy). Figure 5 illustrates the different degrees of exposure to the effects of COVID-19 on regional vulnerability, depending on the level of poverty. The ICC results are grouped into quartiles according to the number of regions. The regions in which socio-economic vulnerability would be most exposed to the effects of COVID-19 (fourth quartile, between 61.1% and 84.3%) would be all of Portugal, Greece, Croatia, Estonia, Latvia, and Lithuania; a large portion of the territory of Spain, Romania and Bulgaria; southern Italy and the eastern regions of Poland. It is worth noting that much of the territory of Sweden and Finland and a region of Ireland that occupied a better position in the SEVI would be among the regions most exposed to the covariate effects. On the other hand, most of the regions of Denmark, Slovakia, the Czech Republic, the Netherlands, Hungary, northern Italy, southern Finland and one of the three regions of Ireland register values in the first quartile (between 30.5% and 53.4%).

## Conclusions and Discussion

The EU Cohesion Policy for the period 2021–2027 focuses on five goals for the EU to become smarter, greener, more connected, more social and closer to citizens. However, a macroeconomic index (per capita GD) is proposed as the predominant criterion for classifying the regions and allocating the Structural Funds. We hypothesise that it is possible to consider new complementary criteria that better reflect citizens’ quality of life. This approach is especially important because the COVID-19 has exposed the vulnerabilities within the EU in all the domains: jobs, education, economy, welfare systems and social life (European Commission, 2021c). On this basis, we have built a composite socio-economic vulnerability index (SEVI) for each of the 233 NUTS-2 of the EU in 2017 that synthesises the information on fragility and resilience factors in order to achieve the objectives of the 2021–2027 Cohesion Policy. The idea is that the higher the value of SEVI, the greater the difficulty in achieving these objectives compared to the rest of the regions.

By implementing the SEVI as an allocation mechanism of the Structural Funds rather than GDP per capita as proposed by the EU, and with an equivalent budgetary effort in terms of the benefited population, we obtain remarkable differences. Our main findings are that a large number of regions in Italy and Spain and some in Portugal, France and Greece which exceed the limit in terms of GDP should be in the group of the most benefited regions according to their socio-economic vulnerability. On the contrary, regions in Member States of the previous eastern Europe, which are historically characterised by low levels of GDP, reach relative positions of less socio-economic vulnerability in our multidimensional approach. These differences in the maps of priority regions could be a source of debate surrounding the introduction of new game rules for the EU Cohesion Policy, especially in the current context of economic and social changes where public policies should prioritise improving citizens’ quality of life.

In a second stage, we study the effects of COVID-19 on regional vulnerability since it is foreseeable that the pandemic will trigger inequalities and increase poverty levels (Fetting, 2020; Giovannini et al., 2020; Shek, 2021; United Nations, 2020a, b). The question is whether all regions will be equally exposed to COVID-19 in terms of their socio-economic vulnerability. To answer this question, we analyse both the idiosyncratic and covariate shocks that COVID-19 might represent by estimating multilevel models. Our findings indicate that increases in government expenditures in education and improving political stability would reduce the regional vulnerability or foster the capacity for resilience. On the other hand, increases in regional monetary poverty would be associated with increased vulnerability, causing bigger growth in the regions with a higher level of vulnerability. Even though regions with a larger SEVI would be the most exposed to the effects of COVID-19, it is remarkable that much of the territory of Sweden and Finland and the region of Ireland that ranked highest in the SEVI would be among the most exposed to the covariate effects. These results might have public policy implications; for example, to inform on how to distribute the European COVID-19 Recovery Funds.

The multidimensional character of our proposal, the study of regions’ factors of vulnerability, fragility and resilience, fits into the mainstream view of economists and policymakers who argue that associating the notion of economic and social progress to a one-dimensional variable of economic activity, such as GDP or income, is debatable (Fetting, 2020; O’Donnell et al., 2014; Sánchez et al., 2018; Sánchez & Ruiz-Martos, 2018; Stiglitz et al., 2018). Our proposal is also in line with two plans or strategies that the European Commission has recently approved to continue advancing in the double green and digital transition and to recover from the COVID-19 crisis: the European Pillar of Social Rights Action Plan (European Commission, 2021c) and the *2020 Strategic Foresight Report* (European Commission, 2020b). The objective of both plans is to promote resilience through the EU institutions so that Europe will recover faster and emerge stronger from the COVID-19 crisis and future crises. A key aspect is that the priorities identified in both plans must be taken into account in all EU policymaking, including the 2021–2027 Cohesion Policy.

In this paper, we have argued that, in terms of Cohesion Policy, there is still room to go ‘beyond GDP’ and consider, in financial terms, the vulnerability and resilience factors that determine people’s well-being. The two previous initiatives or strategies lead us to be hopeful and to think that the EU will continue to advance on the ‘beyond GDP’ path by strengthening the principles of a social market economy. Likewise, the type of exercise carried out in the second stage of this paper can be useful to stimulate discussions regarding the guidelines on how to increase the resilience and reduce the fragility of the regions in order to cope with unforeseen shocks.

Lastly, we would like to point out that our approach to study socio-economic vulnerability differs from most of the studies carried out in this field in the following regards. Firstly, a large number of studies focus on defining vulnerability as the likelihood that, at a given time in the future, an individual will have a level of welfare (income, consumption, poverty, etc.) below some threshold established in a ‘normative’ way. In contrast, under our methodological approach, the choice of normative or arbitrary thresholds is not required, thus overcoming one of the main criticisms of methods involving the elaboration of composite indicators and vulnerability analysis (see, for instance, Dutta et al., 2011; Gallardo, 2018; Nájera & Gordon, 2019; Povel, 2015). Secondly, we do not study risk by estimating the probability of occurrence of future events (for instance via probit/logit models) because our dependent variable in the multilevel models (SEVI) is expressed in a metric. Therefore, to express it as a categorical variable, it would be necessary to collapse the values into two categories, which means that a ‘normative’ threshold would have to be set to establish the limit of the two values, as well as assuming an unnecessary loss of information.

## Figures and Tables

**Fig. 6:**
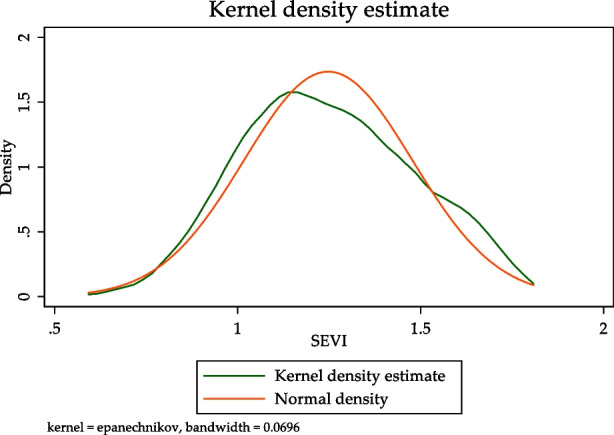
Density function of socio-economic vulnerability index (SEVI) of EU regions (N = 233)

## Appendix

Tables

**Table 5:** Definitions and sources of the single indicators to build SEVI

Name and definition	Source	Geographical level	Date
Early leavers from education and training denotes the percentage of the population aged 18 to 24 having attained at most lower secondary education and not being involved in further education or training	Eurostat, Educational attainment level and transition from education to work (based on EU-LFS) (edat_lfse_04)	NUTS-1 for some regions in AT, DE, FI, FR, IT, PL, UK. NUTS-2 for all other countries	Average 2016–2017
PM2.5. Mean population exposure to fine particles PM2.5. Micrograms per cubic metre	OECD, Environment Database – Exposure to PM2.5	NUTS-2, own elaboration	Average 2016–2017
Senior people. Percentage of senior people in population (75 years or over)	Eurostat, Population change – Demographic balance and crude rates at regional level (demo_r_pjangroup)	NUTS-2	Average 2016–2017
Male unemployment. Unemployment rate % from 20 to 64 years (male)	Eurostat, Regional labour market statistics (lfst_r_lfu3pers)	NUTS-2	Average 2016–2017
Female unemployment. Unemployment rate % from 20 to 64 years (female)	Eurostat, Regional labour market statistics (lfst_r_lfu3pers)	NUTS-2	Average 2016–2017
Youth unemployment rate % from 15 to 24 years (female + male)	Eurostat, Regional labour market statistics (lfst_r_lfu3pers)	NUTS-1 for some regions in AT, DE, FI, HU, LT, PL, PT, UK. NUTS-2 for all other countries	Average 2016–2017
Migrant. Crude rate of net migration plus statistical adjustment. Difference between the crude rate of population change and the crude rate of natural change; that is, net migration is considered as the part of population change not attributable to births and deaths expressed per 1,000 inhabitants. Only positive rates are considered, otherwise zero is assigned	Eurostat, Population change – Demographic balance and crude rates at regional level (demo_r_gind3)	NUTS-2	Average 2016–2017
Assault & crime. Number of deaths by assault and homicide divided by population and then multiplied by 100,000 (crude death rate)	Eurostat, Causes of death – crude death rate by NUTS-2 region of residence (hlth_cd_acdr2)	NUTS-2	Average 2016–2017
R&D business. Intramural R&D expenditure Business enterprise sector (percentage of gross domestic product)	Eurostat, Statistics on research and development (rd_e_gerdreg)	NUTS-1 for some regions in BE, LT, NL, PL. NUTS-2 for all other countries	Average 2015–2016, except: BE 2014–2015; AT, DE, EL, IE 2015; NL 2014; FR 2013
R&D state. Intramural R&D expenditure Government sector + higher education sector (percentage of gross domestic product)	Eurostat, Statistics on research and development (rd_e_gerdreg)	NUTS-1 for some regions in BE, LT, NL, PL. NUTS-2 for all other countries	Average 2015–2016, except: BE 2014–2015; AT, EL, IE, PL, SE 2015; NL 2014; FR 2013
Tertiary education. Individuals aged 25–64 who successfully completed tertiary education (levels 5–8 ISCED 2011) over the population with the same age (In %)	Eurostat, Educational attainment level and transition from education to work (based on EU-LFS) (edat_lfse_04)	NUTS-2	Average 2016–2017
Human resources in technology. Persons employed in science and technology as percentage of active population	Eurostat, Human Resources in Science & Technology (hrst_st_rcat)	NUTS-2	Average 2017–2018
Registered community designs. Number of registered community designs per billion GDP purchasing power standards	Eurostat, Community design (ipr_dr_reg)	NUTS-2	Average 2015–2016
Internet. Percentage of households with internet access at home	Eurostat, ICT usage in households and by individuals (isoc_r_iacc_h)	NUTS-1 for some regions in DE, EL, PL, UK. NUTS-2 for all other countries	Average 2017–2018
E-Administration. Percentage of individuals who used the Internet for interaction with public authorities (last 12 months)	Eurostat, ICT usage in households and by individuals (isoc_r_gov_i)	NUTS-1 for some regions in DE, EL, PL, UK. NUTS-2 for all other countries	Average 2017–2018
GDP-Gini. Regional gross domestic product (GDP) purchasing power standard per inhabitant adjusted by the country Gini index of disposable household income [GDP per capita*(1-Gini index)]. Disposable household income includes: all income from work (employee wages and self-employment earnings), private income from investment and property, transfers between households, and all social transfers received in cash, including old-age pensions	Eurostat, Regional economic accounts (nama_10r_2gdp) and Income and living conditions (ilc_di12)	NUTS-2	Average 2016–2017

^5^,

**Table 6:** Selection of single indicators to build the socio-economic vulnerability index of European regions

**Fragility indicators**	**European Union plans, strategies and projects**
Early leavers	European 2020 Strategy (European Commission, 2010) Prototype dashboard for social and economic resilience (European Commission, 2020b) The European Pillar of Social Rights Action Plan (European Commission, 2021c)
PM2.5	Prototype dashboard for the green resilience (European Commission, 2020b) Regional Innovation Scoreboard 2021 (European Commission, 2021a)
Senior people	Prototype dashboard for social and economic resilience (European Commission, 2020b) The European Pillar of Social Rights Action Plan (European Commission, 2021c)
Male unemployment	Prototype dashboard for social and economic resilience (European Commission, 2020b) The European Pillar of Social Rights Action Plan (European Commission, 2021c)
Female unemployment	Prototype dashboard for social and economic resilience (European Commission, 2020b) The European Pillar of Social Rights Action Plan (European Commission, 2021c)
Youth unemployment	Prototype dashboard for social and economic resilience (European Commission, 2020b) The European Pillar of Social Rights Action Plan (European Commission, 2021c)
Migrant	The European Pillar of Social Rights Action Plan (European Commission, 2021c) Allocation of Cohesion Policy Funding (European Court of Auditors, 2019)
Assault & crime	Prototype dashboard for social and economic resilience (European Commission, 2020b)
**Resilience indicators**	**European Union plans, strategies and projects**
R&D business	European 2020 Strategy (European Commission, 2010) Prototype dashboard for social and economic resilience (European Commission, 2020b) Regional Innovation Scoreboard 2021 (European Commission, 2021b)
R&D State	Prototype dashboard for social and economic resilience (European Commission, 2020b) Regional Innovation Scoreboard 2021 (European Commission, 2021b)
Tertiary education	European 2020 Strategy (European Commission, 2010) Regional Innovation Scoreboard 2021 (European Commission, 2021b)
Human resources in technology	Regional Innovation Scoreboard 2021 (European Commission, 2021b)
Registered community designs	Regional Innovation Scoreboard 2021 (European Commission, 2021b) Cohesion Policy (European Commission, 2018)
Internet	Prototype dashboard for the digital resilience (European Commission, 2020b)
E-Administration	Prototype dashboard for the digital resilience (European Commission, 2020b)
GDP-Gini	Cohesion Policy (European Commission, 2018) Allocation of Cohesion Policy Funding (European Court of Auditors, 2019)

6,

**Table 7:** Definitions and sources of the variables for multilevel analysis

Name and definition	Source	Geographic level	Date
Poverty. Percentage of inhabitants with disposable income below the 60% of the country’s median disposable income	Eurostat, Income and living conditions (ilc_mddd21)	Country level for BE, DE and FR, NUTS-1 for PL and NUTS-2 for all other countries	2018
Education. Government expenditure in education (percentage of gross domestic product)	Eurostat, General government expenditure by function (COFOG) (gov_10a_exp)	Country (general government)	Average 2017–2018
Stability. Estimator of governance that measures the perceptions of political stability and absence of politically-motivated violence, including terrorism. Ranges from approximately -2.5 weak to 2.5 strong governance performance	OECD, Worldwide Governance Indicators	country	2018

^7^

Figure 6

## Footnotes

1 Nomenclature of territorial units for statistics level 2 (NUTS-2) is the classification used in regional statistics and funding allocation which subdivides Member States into regions according to existing national administrative subdivisions and the population thresholds from 800,000 to 3 million inhabitants. The EU Cohesion Policy is designed and monitored at NUTS-2 level.
